# The chlorophyll fluoroscope, a device to observe the in vivo emission of chlorophyll fluorescence for teaching and demonstration purposes

**DOI:** 10.1007/s11120-025-01150-9

**Published:** 2025-04-15

**Authors:** João Serôdio

**Affiliations:** CESAM – Centre for Environmental and Marine Studies and Department of Biology, University of Aveiro, Campus de Santiago, Aveiro, 3810-193 Portugal

**Keywords:** Chlorophyll fluorescence, Teaching, Science demonstration, Plant leaves, Macroalgae

## Abstract

In vivo chlorophyll fluorescence has long been known to be intimately related to photosynthesis, being the basis of widespread and sophisticated instrumentation. Although easily observed in plant extracts or using epifluorescence or confocal microscopes, chlorophyll fluorescence is seldom observed in macroscopic samples, such as plant leaves of macroalgae thalli. This work presents a ‘chlorophyll fluoroscope’, a device that allows the direct observation of the fluorescence emitted in vivo by large samples. The chlorophyll fluoroscope is easy to construct and operate, comprising inexpensive 3D-printed parts and off-the-shelve components. It is primarily intended for use in teaching and science demonstration events while having the potential to interest those who are aware of chlorophyll fluorescence yet often have never observed the phenomenon.

**Supplementary Information:**

The online version contains supplementary material available at 10.1007/s11120-025-01150-9.

## Introduction

One of the hardest things when teaching plant physiology is convincing students that all plants, algae and other photosynthetic organisms, emit red light – chlorophyll fluorescence – when exposed to sunlight. They are told that this intriguing and often unheard-of type of radiation is of the utmost importance for studying photosynthesis, as it is the basis of a whole set of complex photophysiological parameters, and of a panoply sophisticated instruments specifically designed for its measurement. And yet, despite being well within the spectrum detected by our eyes, its observation is difficult: it is too faint to be distinguished from ambient light – ironically, necessary to cause its emission it in the first place. This conundrum is usually resolved by preparing plant pigment extracts, where the yield of chlorophyll fluorescence is greatly enhanced. With proper focused and unilateral illumination against a dark background, an intense deep red color can be easily observed in an otherwise green liquid. Although this demonstration is novel and impressive to most observers, it is a poor substitute for observing the real in vivo emission by intact plant leaves, the existence of which still requires a leap of faith.

The in vivo emission of chlorophyll fluorescence can indeed be observed, but it requires a combination of (i) illumination with intense monochromatic (non-red, preferably blue) light, (ii) selective filtering of the emitted red radiation, and (iii) elimination of ambient light. These conditions are routinely achieved using epifluorescence or confocal microscopy, techniques that allow the observation of what is referred to, in this context, as ‘chlorophyll autofluorescence’ (García-Plazaola et al. 2015). These techniques naturally restrict the observation to microscopic objects, individual cells, and optically thin tissues, explaining why images of chlorophyll fluorescence of cells are very common, while equivalent images of macroscopic objects like plants, macroalgae or lichens are rare (Stirbet et al. 2014; Lagorio et al. 2015).

This work presents a simple device, named the ‘chlorophyll fluoroscope’^1^, designed to enable the direct observation of chlorophyll fluorescence in living plants and other macroscopic photosynthetic organisms. The device is easy to build, utilizing 3D-printed parts and off-the-shelf inexpensive and readily available components. The chlorophyll fluoroscope is portable and easy to operate, making it particularly suited for teaching and science demonstration events. In vivo chlorophyll fluorescence can be readily observed directly or acquired using any camera, including mobile phone cameras, enhancing its accessibility and practicality for students and non-academic users.

## Rationale

To enable the observation of chlorophyll fluorescence, the fluoroscope must be capable of inducing a strong fluorescence emission and filtering out any radiation other than emitted fluorescence. The proposed chlorophyll fluoroscope achieves this by combining: (i) a source of intense monochromatic blue light, which is highly absorbed by chlorophyll molecules and intense enough to trigger a strong fluorescence emission, while being monochromatic enough to allow reflections to be filtered out; and (ii) a long-pass optical filter, which filters out the blue light reflected from the sample while allowing red chlorophyll fluorescence to reach the observer’s eyes or camera.

**Fig. 1:**
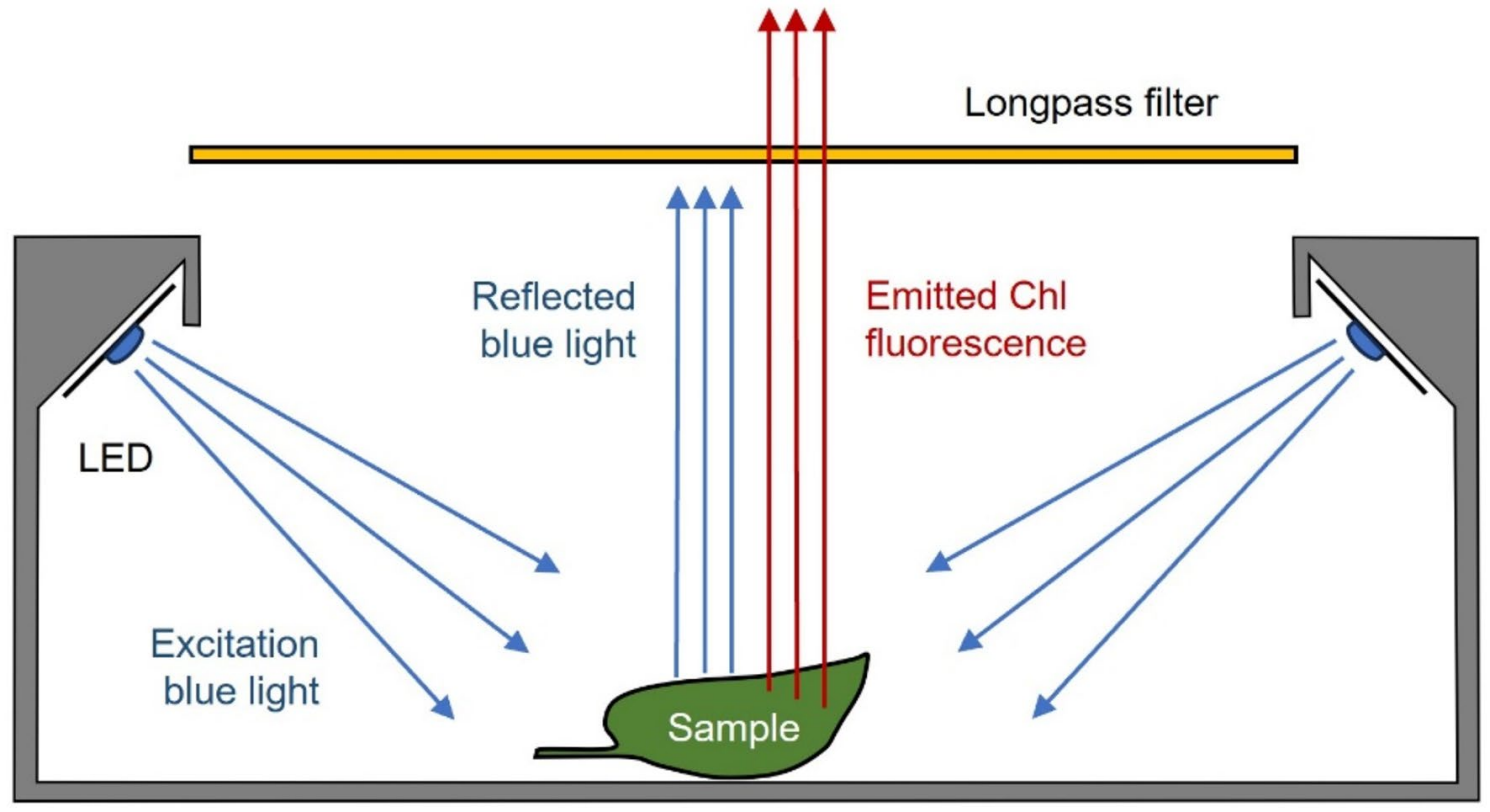
Schematic of the chlorophyll fluoroscope showing the relative position of the LEDs providing intense blue excitation light, the sample and the long-pass filter, and how it prevents the passage of the reflected blue light but not of the chlorophyll fluorescence emitted by the excited sample

The main part of the fluoroscope is a light-shielding housing, designed to keep the samples from exposure to ambient light and facilitate the observation of the faint chlorophyll fluorescence (Fig. 1). The housing is completely closed, except for an observation opening on the top, through which the samples can be viewed. Inside, two arrays of blue LEDs are positioned to face downwards, directing light toward the central area where the samples are placed for observation, while preventing the direct exit of excitation light through the observation opening. The long-pass filter is used externally, between the samples and the observer’s eyes (Fig. 1).

## Components

Excitation light was provided by 9 high-power 5 W star ‘royal blue’ LEDs, mounted on heat dissipative Printed Circuit Boards (https://www.ebay.com/itm/254712798354). To ensure constant current the LEDs were connected in series and powered by an LED driver (25–40 V, 1000 mA; https://www.ebay.com/itm/394417592862). Both the LEDs and the LED driver can be easily purchased online or in shops specialized in electronics.

**Fig. 2:**
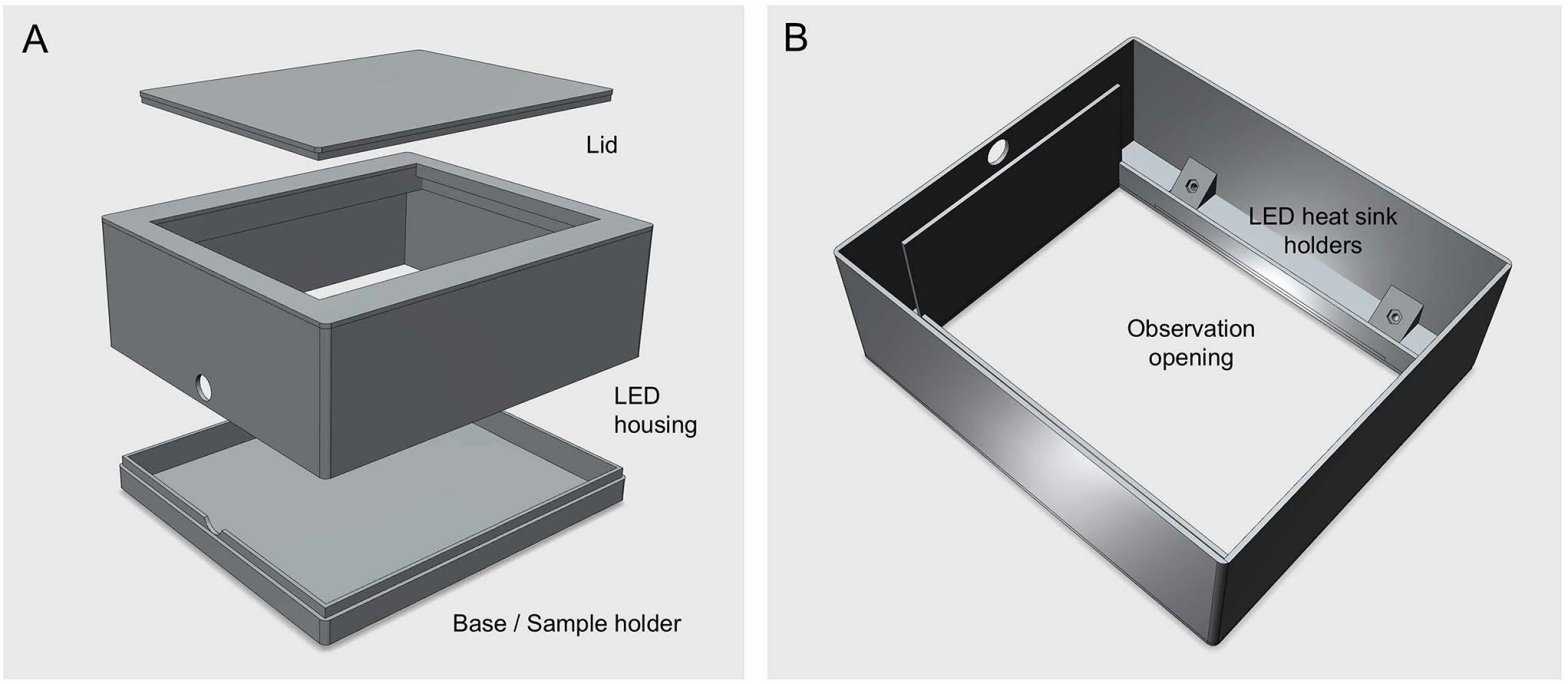
Exploded view of the fluoroscope showing its main parts. **A** Main housing contains LEDs, the removable base where samples are positioned for observation, and the top lid to ensure dark adaptation of the samples. **B** Bottom view, showing the location of the LEDs and respective fixing holes. The shape and size of the observation opening was designed to hold an optional square long-pass filter. All parts were 3D printed in black matte PLA to minimize ambient light reflections inside the housing. STL files are provided as Supplemental material

The chlorophyll fluoroscope was built with 3D-printed parts (Fig. 2), using matte black filament (PLA Matte, Bambu Lab, GmbH, Frankfurt am Main, Germany) to minimize reflections. The parts were printed on a Bambu Lab P1P 3D-printer, but they can be printed using any commercial 3D-printer and any type of black filament. The LED driver was mounted on a separate housing, also 3D-printed. The observation opening can be closed with a dedicated lid to allow samples to dark adapt before the observation. All STL (Stereolithography files used by 3D printers) files are available as supplemental material. Although the fluoroscope is meant to be used by applying short periods of illumination (see below), the array of LEDs can generate significant amounts of heat that can potentially damage the PLA-made housing. To prevent excessive heating, the LEDs were mounted on heat dissipating aluminum plate (170 × 20 × 3 mm) using double sided thermal conductive tape. The aluminum plates were fixed to the housing using 16 mm M3 screws that were attached to M3 nuts inserted and glued to holders in the housing, allowing the plates to remain at 5 mm from the housing parts to allow heat dissipation. One thermal switch (KSD301 50ºC normally closed) was connected in series to the LED array to automatically cut the circuit in the case of excessive heating. To further eliminate reflections of excitation light inside the light-shielding housing, the inner walls and bottom were painted with a super-black coating absorbing 99.9% of incident light (Musou Black Acrylic Paint; www.musoublack.com). Good results are also obtained by covering the bottom of the housing with super-black fabric (Kiwami Fabric; www.musoublack.com). The application of this coating improves the observation but is not strictly necessary. As long-pass filter, an inexpensive handheld dental shield plate (e.g. www.ebay.com/itm/325403324342) was used. It has large dimensions and a holder that facilitates its manipulation and the sample observation.

## Mounting and testing

**Fig. 3:**
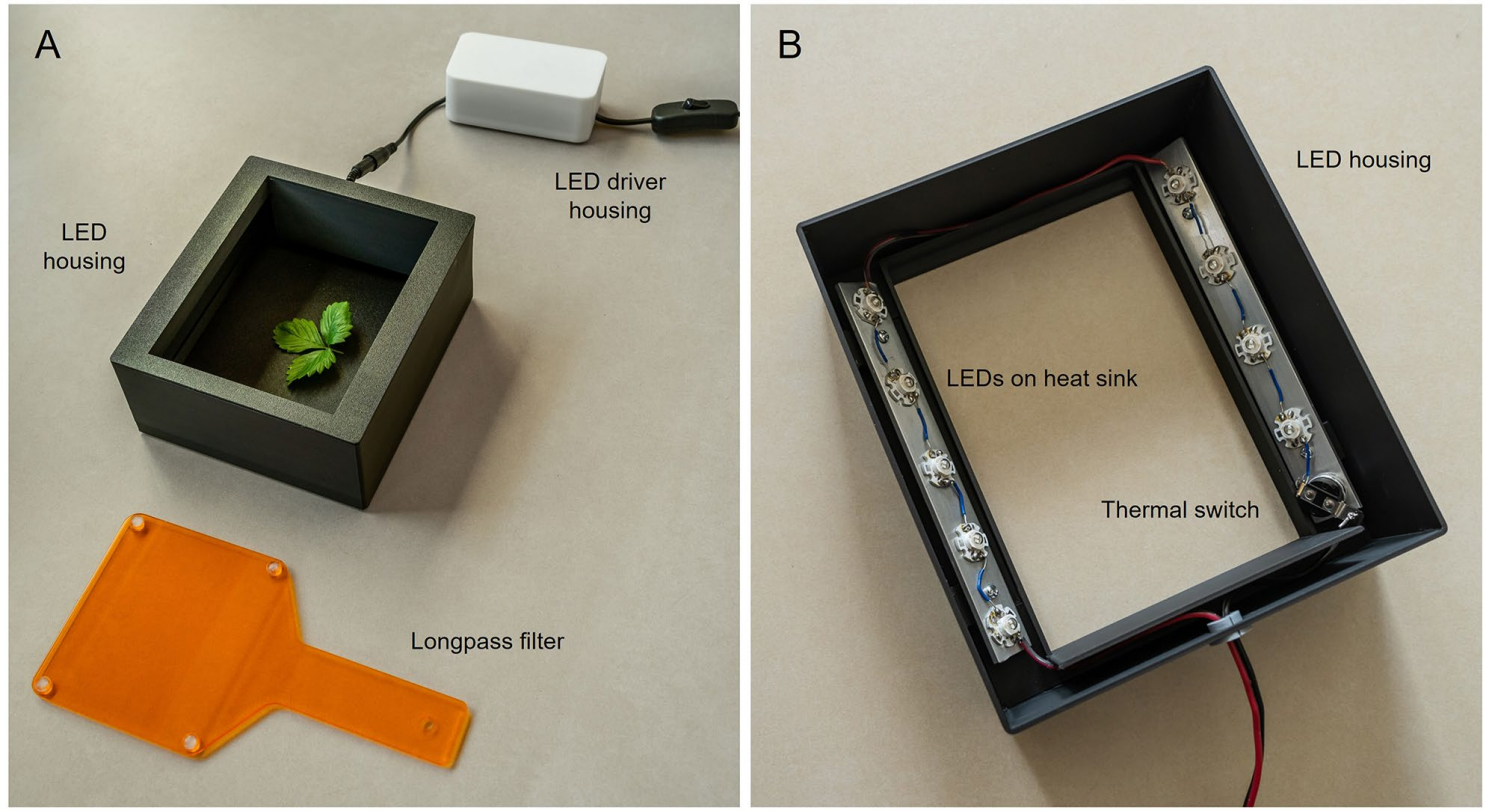
Photographs of the chlorophyll fluoroscope showing (**A**) the LED housing with plant leaves inside, the LED driver housing and attached inline switch, and the long-pass filter, and (**B**) the positioning of the LEDs and thermal switch inside the LED housing, mounted on aluminum heat-dissipating plates

The construction of the fluoroscope requires simple steps: (i) soldering the LEDs and the thermal switch in series using 1–2 cm wires to connect them; (ii) connect the LED array to the LED driver and the LED driver to a standard 110–220 V electrical cable, preferably with an inline switch; (iii) fixing the heat dissipating aluminum plates to the inner face of the housing with M3 screws and nuts; (iv) fixing the LEDs and the thermal switch to the aluminum plates using double sided thermal conductive tape. An overview of the finished system is shown in Fig. 3. The total price of the system can be kept below €60.

**Fig. 4:**
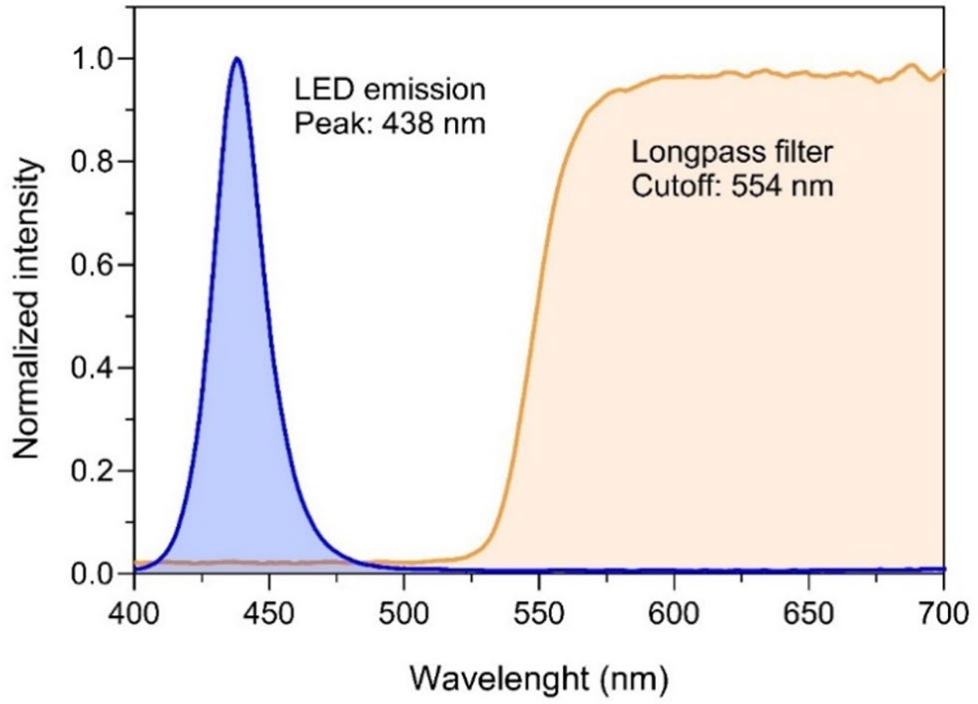
Spectral properties of the LEDs (emission spectrum; blue) and of the long-pass filter (transmission spectrum; orange) used in the chlorophyll fluoroscope

The blue LEDs provided adequately intense and monochromatic excitation light (Fig. 4). Peak emission and full width at half maximum (FWHM) were at 438 nm and 22 nm, respectively. PAR irradiance at the level of samples reached above 500 µmol photons m^-2^ s^-1^. The handheld long-pass filter eliminated the reflected blue excitation light, allowing the transmission of the emitted red chlorophyll fluorescence with minimal attenuation (3.4% for wavelengths > 600 nm). The irradiance and spectra of the excitation light were measured using a spectroradiometer (SpectraPen Mini, Photon Systems Instruments, Drásov, Czech Republic), positioned at the same position of the samples inside the light-shielding box. The same spectroradiometer was used to measure the transmission spectrum of the long-pass filter. The transmission spectrum of the longpass filter was determined by the ratio between irradiance (µmol m^-2^ s^-1^ nm^-1^) measured for each wavelength (400–700 nm) by the sensor exposed to direct sunlight and covered with the longpass filter. The resulting spectrum was smoothed using a 5-point moving average.

## Usage

As chlorophyll fluorescence is in any case a rather dim light, observation should be done in a darkened room, under low ambient light. Better results are obtained when holding the filter closer to the eyes rather than close to the housing. Alternating the observation with and without the filter is also a good way to perceive the origin and distribution of fluorescence in the samples under observation. To increase the emission of chlorophyll fluorescence, as well as its deep red color, it is recommended that samples are dark acclimated for 10–15 min before observation. This can be done using the fluoroscope as a dark acclimation chamber, using the lid to prevent ambient light from reaching the samples inside. It is preferable to turn on the LEDs for short periods as long exposures will cause a decrease in chlorophyll fluorescence emission due to quenching (see below). Despite the measures put in place to prevent excess heating, the use of the fluoroscope in the classroom environment must be always supervised.

The fluoroscope is meant for direct observation (a much more rewarding experience than by other means), but the emission of chlorophyll fluorescence can be easily documented using photography or video. For this article, the detection and observation of chlorophyll fluorescence using the proposed fluoroscope, is illustrated with images captured using a full-frame digital camera (Sony α7 II) mounted on a tripod. However, very good results can be achieved with any mobile phone camera, a popular option in the classroom or science demonstration activities. Optionally, a phone holder and small tripod can be used to better observe the fluorescence emission and easily capture images.

To better illustrate the potential of the fluoroscope, images of chlorophyll fluorescence were compared to conventional images produced by illuminating the same samples with white light (halogen lamp, no filter applied). The images shown were only minimally edited using Adobe Lightroom, exclusively to eliminate distracting features (dust, reflections) in the background.

## Biological material

The use of the chlorophyll fluoroscope was illustrated by using leaves of common plant species and marine macroalgae. The plant leaves were collected in and around the *campus* of the University of Aveiro and the macroalgae were collected at Granja Beach, Portugal, in December 2024. Samples were chosen to demonstrate different aspects affecting the emission of chlorophyll fluorescence that are not necessarily visible to the naked eye: the presence of leaf parts lacking chlorophyll *a*, leaf senescence and loss of chlorophyll *a*, the presence of pigments other than chlorophyll *a* emitting non-red fluorescence (red macroalgae), and chlorophyll fluorescence quenching.

## Observations

**Fig. 5:**
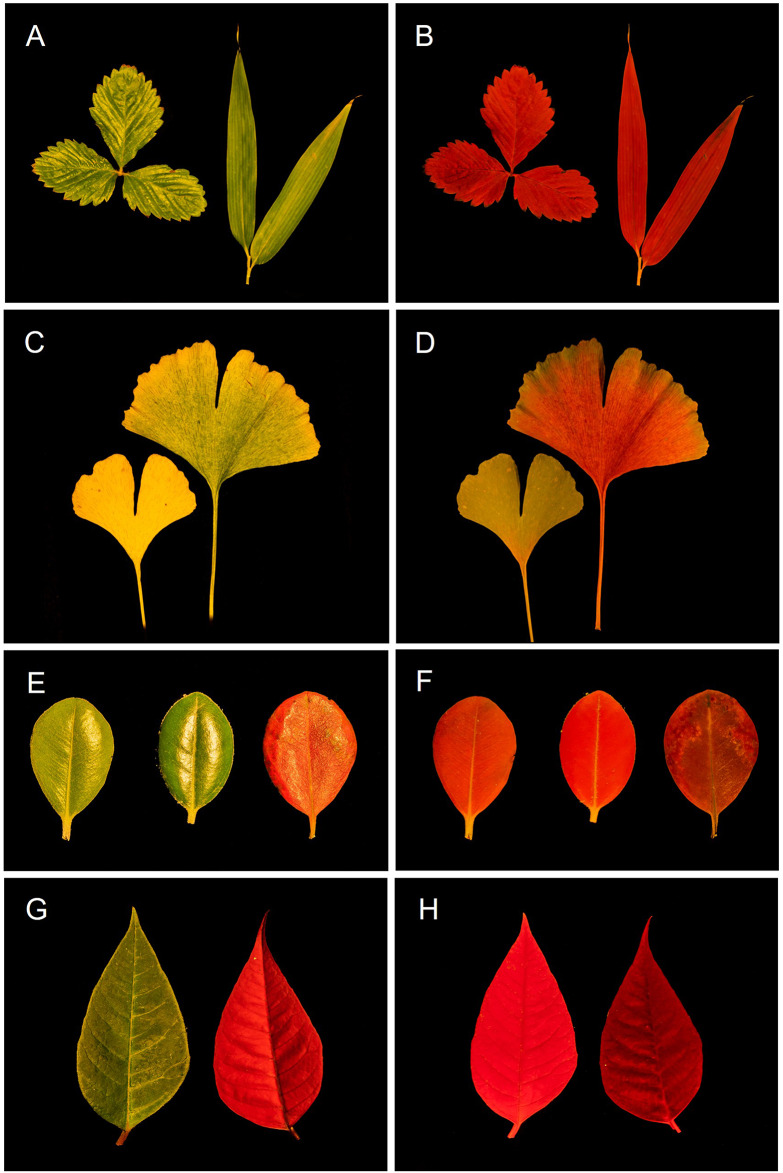
Photographs of plant leaves, as illuminated by white light (**A**, **C**, **E**, **G**), and corresponding images of chlorophyll fluorescence emission, as produced by the fluoroscope (**B**, **D**, **F**, **H**). **A**, **B** Strawberry (*Fragaria* × *ananassa*) and bamboo (*Bambusa nana*). **C**, **D***Ginkgo biloba* (senescent yellowing and partially green leaves). **E**, **F***Metrosideros excelsa* (young, mature and senescent leaves). **G**, **H***Euphorbia pulcherrima* (green and red leaves)

**Fig. 6:**
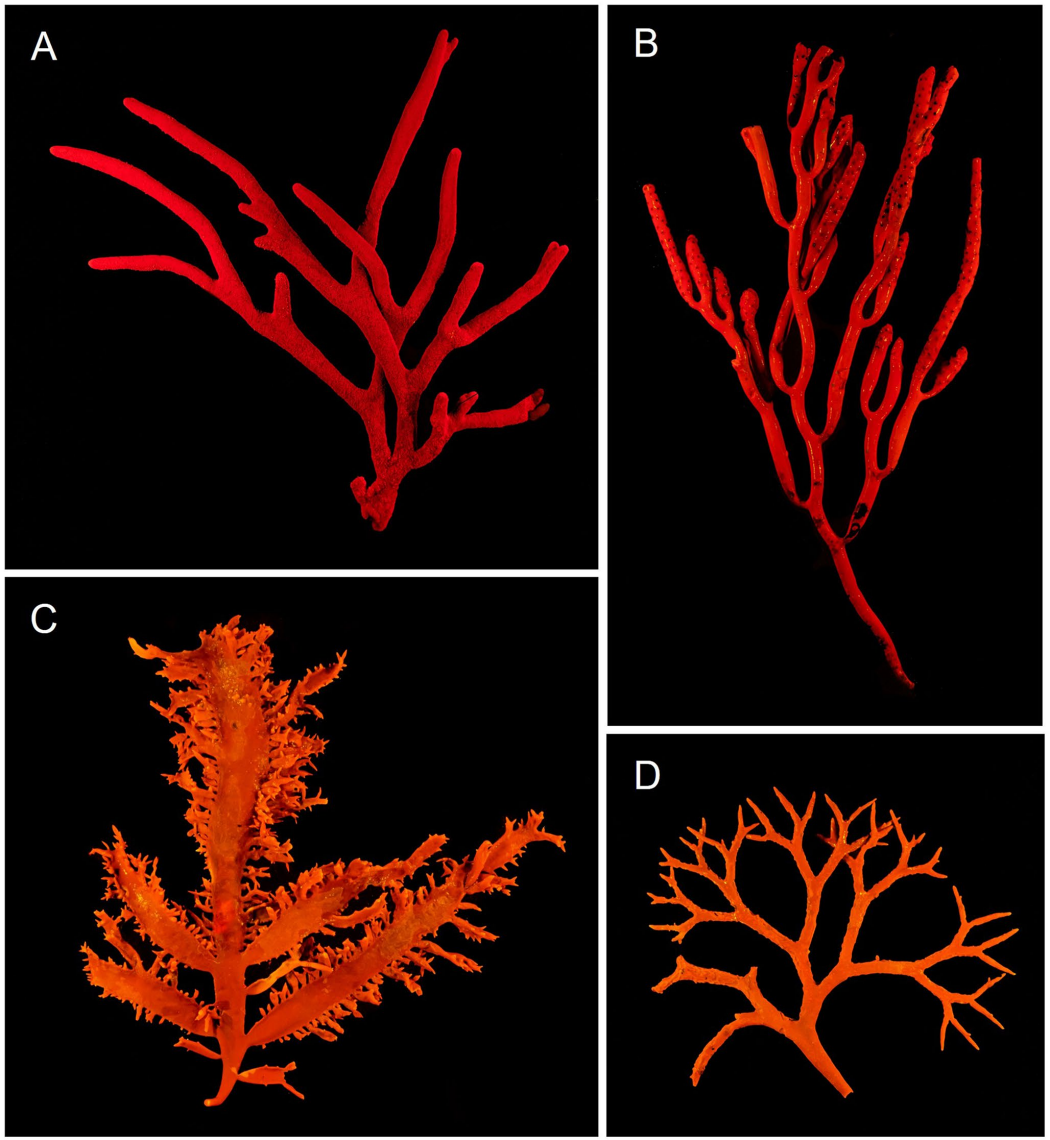
Photographs of chlorophyll fluorescence emission by macroalgae. Red fluorescence by *Codium fragile* (Chlorophyta; **A**) and *Bifurcaria bifurcata* (Phaeophycea; **B**), and orange fluorescence by *Chondracanthus teedei* (Rhodophyta; **C**) and *Gigartina pistillata* (Rhodophyta; **D**)

**Fig. 7:**
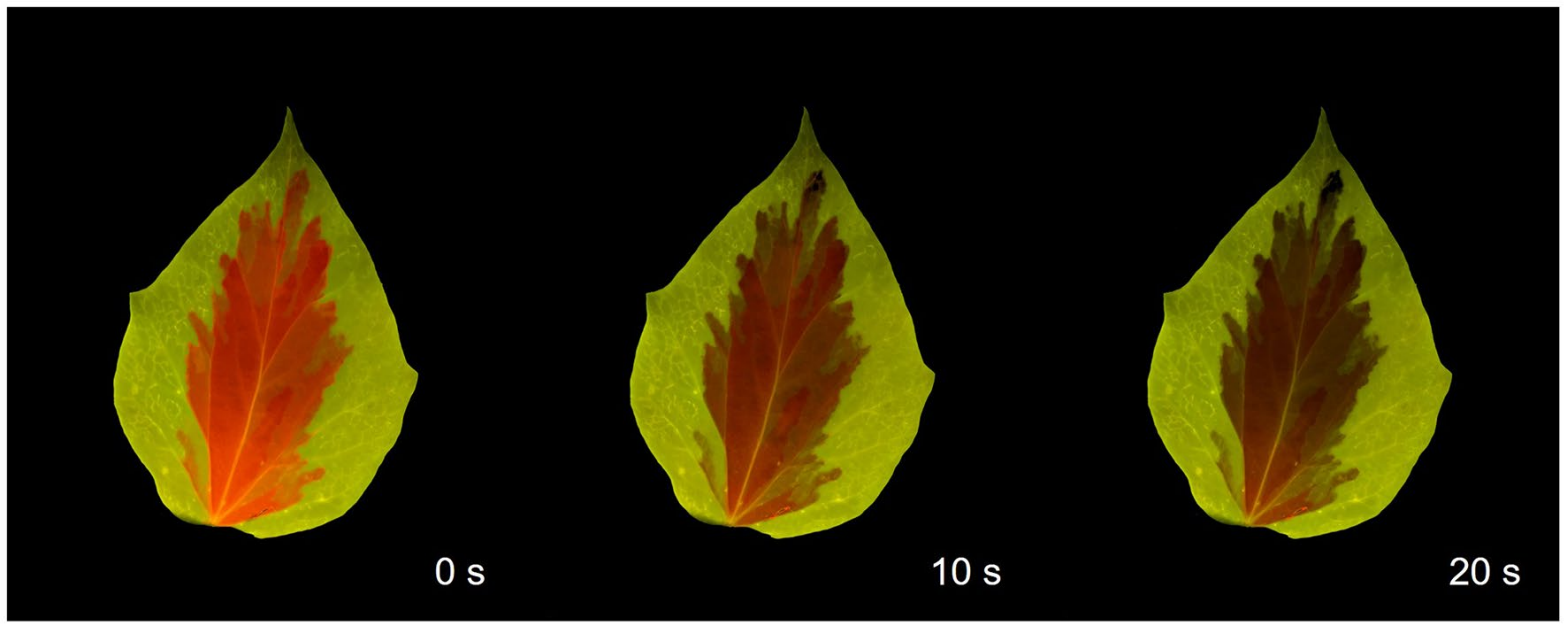
Quenching of chlorophyll fluorescence as observed in a dark-acclimated leave of ivy (*Hedera helix*) as exposed to bright blue light. Times refer to exposure to blue light

Figures 5, 6, and 7illustrate the use of the chlorophyll fluoroscope to observe the in vivo emission of chlorophyll fluorescence in macroscopic photosynthetic samples. It must be emphasized that while the chlorophyll fluorescence images of leaves and macroalgae are impressive, they do not fully capture the sense of beauty experienced when observed in person.

Figure 5 highlights the striking difference observed when comparing conventional photographs of leaves illuminated by white light (and no filter applied; left-hand panels) to the chlorophyll fluorescence images (right-hand panels). A unique feature of the chlorophyll fluorescence images as compared to conventional photographs, particularly evident in pairs A, B and E, F, is the absence of any glow, as the long pass filter removes all incident light reflected by the glossy epidermis of the leaves. This aspect can be emphasized as an indication that the red color is produced by the leaf and is not reflected light.

Figures 5C-H illustrate the various ways in which different chlorophyll *a* contents affect the observation of fluorescence emission. Figures 5(C, D) demonstrate that the yellowing of senescent *Ginkgo biloba* leaves, as seen under white light, is accompanied by a significant decrease in chlorophyll fluorescence emission, due to the reduction of chlorophyll *a* content. The fully senescent leaf (Fig. 5C, D; left leaf), which appears completely yellow under white light, shows no fluorescence emission. In contrast, the partially senescent leaf (Fig. 5C, D; right leaf) retains some green areas, corresponding to regions still emitting visible chlorophyll fluorescence.

Figures 5E-F illustrate how the increase in chlorophyll content from young to mature leaves of *Metrosideros excelsa* (left vs. center leaves) is followed by an increase in the red signal. The senescence of *Metrosideros* leaves causes the appearance of a bright red color (Fig. 5E, right leaf), due to the accumulation of anthocyanins, with the role of reducing the excitation pressure on PSII and the risk of photodamage (Hoch et al. 2003). Interestingly, despite the red color observed under white light, the chlorophyll fluorescence signal in this case lower, due to the decrease of chlorophyll *a* content or functional photosystems II (PSII) or the epidermal screening by anthocyanins. A similar phenomenon occurs in *Euphorbia pulcherrima* (Fig. 5G, H), which also has leaves that produce anthocyanins (Lozoya-Gloria et al. 2023). These leaves appear bright red under white light (Fig. 5G, right leaf) but emit a much dimmer chlorophyll fluorescence than the leaves without anthocyanins (Fig. 5H; left leaf).

The fluoroscope is also useful for demonstrating the emission of chlorophyll fluorescence by macroscopic photosynthetic samples other than plant leaves. Figure 6 shows examples of images obtained from marine macroalgae of different groups. Green (Chlorophyta) and brown (Phaeophyceae) algae emit a strong red fluorescence similar to that of plants (Fig. 6A, B) but red algae (Rhodophyta) emit orange fluorescence (Fig. 5C-D), which results from the combination of red fluorescence emitted by chlorophyll *a* and yellow fluorescence (570–580 nm) emitted by the pigment phycoerythrin, characteristic of this group (MacColl 1991).

Another physiologically relevant phenomenon that can be demonstrated using the chlorophyll fluoroscope is the quenching of fluorescence emission upon illumination of dark-acclimated samples with bright light (Kautsky effect; (Kautsky and Hirsch 1931; Govindjee 1995)). This effect can be easily illustrated with virtually any type of leaf but is particularly emphasized using leaves with non-chlorophylline parts, such as the variety of *Hedera helix* shown in Fig. 7: the central chlorophyll *a*-containing region of the leaf emits red fluorescence, which rapidly fades away, while the peripheral regions lacking chlorophyll *a* maintain their initial color throughout the exposure to light. The quenching of chlorophyll fluorescence is a distinctive signature of photosynthesis and is of utmost importance when discussing the relation between fluorescence emission and photosynthesis.

**Fig. 8:**
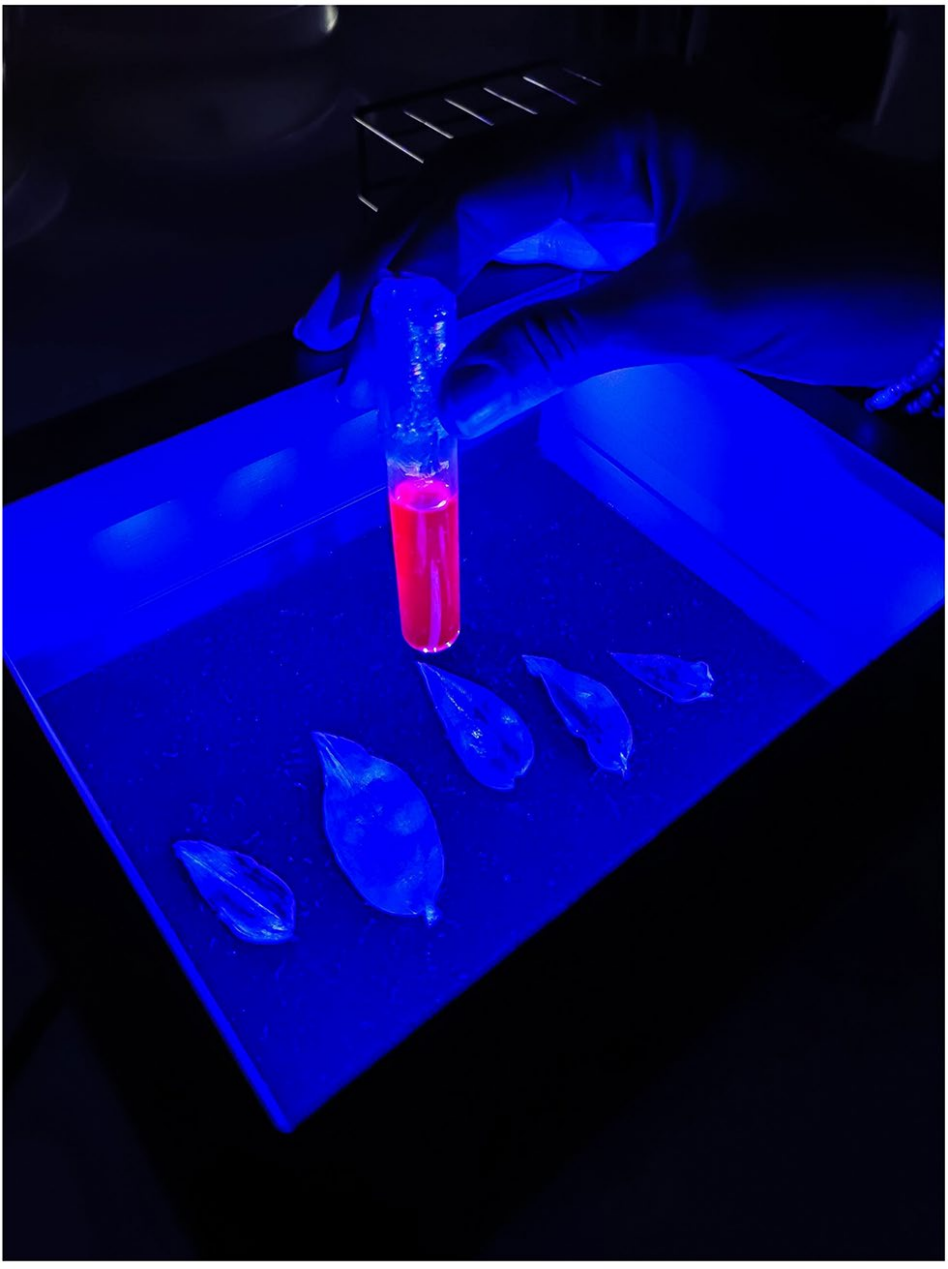
Chlorophyll fluorescence emission by an acetone extract of *Tradescantia zebrina* (tube), as observed without the long-pass filter. Note that the fluorescence emission is much more intense in the extract than in leaves, which is not visible without using the long-pass filter

By providing a strong blue light on a darkened background, the fluoroscope is particularly suitable to observe the chlorophyll fluorescence emission in plant extracts, a demonstration commonly carried out in Plant Physiology classes. As shown in Fig. 8, the fluoroscope enables easy observation of chlorophyll fluorescence emitted by an acetone extract, even without using the long-pass filter to block the reflected excitation blue light. This image also highlights an important observation for interpreting the physiological meaning of chlorophyll fluorescence: that the fluorescence yield is much more intense in an extract than in vivo. This is because in the extract, the thylakoid membranes are dissolved and the pigments (chlorophyll *a* and accessory pigments) can no longer transfer the energy of absorbed photons between them, within the light-harvesting complex antenna, and to the reaction centers, or dissipate absorbed energy as heat. This results in that pigment molecules will dissipate most of the absorbed energy as fluorescence. Table 1 summarizes the various types of observations, and their respective physiological meanings, that can be carried out with the chlorophyll fluoroscope for teaching or science demonstration events.

**Table 1:** Summary of observations, and correspondent interpretation, which can be demonstrated using the chlorophyll fluoroscope to teach key concepts of photosynthesis. In all cases, dark acclimation is recommended before observation. Short: observation immediately after turning on the light. Chl: chlorophyll

Material	Illumination	Observation	Interpretation
Healthy green leaf	Short	Strong red fluorescence	Presence of Chl *a* and functional PSII
		Leaves do not glow	Fluorescence is emitted by the sample, not reflected
Senescent leaf	Short	No fluorescence	No Chl *a* or functional PSII present
Red-colored leaf	Short	Weak fluorescence	Red color under white light is due to anthocyanins that do not fluoresce
Green tuber or fruit	Short	Red fluorescence	Presence of Chl a and functional PSII
Brown macroalgae	Short	Red fluorescence	Contain brown pigments (fucoxanthin) do not fluoresce
Red macroalgae	Short	Orange fluorescence	Combined emission of red and yellow fluorescence, emitted by Chl *a* and phycoerythrin
Healthy green leaf	Prolonged	Fluorescence attenuates over time	The Kaustky effect, light-activation of photosynthesis and gradual quenching of fluorescence
Plant acetone extract	Short	Fluorescence is much stronger than in leaves, observable without the filter	Chl *a* is present but is no longer organized in light-harvesting antennae, quenching processes not effective

## Potential, limitations and alternatives

Chlorophyll fluorescence is central to the teaching of photosynthesis. Not for being a mere curiosity, an anecdotical and inconsequent phenomenon, but because it is a rich source of information about the mechanisms of this complex process. Since the realization of Kaustky and Hirsch (1931) of its extraordinary significance and intimate relationship with the photosynthetic process, chlorophyll fluorescence has been established as a metabolic probe for the in vivo study of photosynthesis (Govindjee 1995), becoming the basis of sophisticated specialized instrumentation – chlorophyll fluorometers – designed to detect and measure it in living organisms in increasingly precise and diverse ways (Falkowski et al. 1986; Schreiber et al. 1986; Klughammer et al. 2024).

However, chlorophyll fluorescence is rarely seen with the naked eye in human-scale photosynthetic samples like leaves, macroalgae, lichens, or corals. The described fluoroscope, though simple in design and construction, produces striking live visualizations of the chlorophyll fluorescence emission in such macroscopic objects. It is easy to operate in a classroom environment and, in my experience, it sparks the interest of even the most reluctant students, representing an invaluable addition to the practical sessions of a Plant Physiology course. Seeing chlorophyll fluorescence in such a straightforward manner also impresses and evokes a sense of awe in those who are familiar with its existence, and even in those who have worked extensively with chlorophyll fluorometers but seldom realize the visual phenomenon behind the data. It serves as a form of ‘ground-truthing’, providing a tangible reality to the abstract numbers and indices generated by the fluorometers.

The main limitation of the present configuration is the relatively small size of the specimens that can be used (10–12 cm in length, maximum). However, this model can be easily upscaled to accommodate larger samples, the main requirement being an increase of the number of LEDs to ensure the induction of easily visible chlorophyll fluorescence. The increase in the number of LED must be accompanied by the adequate upscaling in the capacity of heat dissipation.

In designing the presented fluoroscope, the use of easily accessible and inexpensive components and parts was prioritized. However, similar (or even better) results may be achieved with alternative items. For excitation light sources, any powerful monochromatic blue light source can be used (e.g. SL 3500-B LED Light; Photon Systems Instruments, Brno, Czech Republic). Although these commercially available systems are much more expensive, they have the advantage of illuminating a larger area, making them suitable for observing larger specimens. Regarding the long-pass filter, two options were tested and found equally efficient: protective glasses for visualizing gels (XcitaBlue Viewing Goggles; Bio-Rad, Richmond, California, USA) and color corrective filters (Deep Blue 47 Wratten filter; Kodak, Rochester, New York, USA).

## Electronic supplementary material

Below is the link to the electronic supplementary material.

Supplementary Material 1

Supplementary Material 2

Supplementary Material 3

Supplementary Material 4

Supplementary Material 5

Supplementary Material 6

Supplementary Material 7

Supplementary Material 8

Supplementary Material 9

Supplementary Material 10

Supplementary Material 11

Supplementary Material 12

## Data Availability

No datasets were generated or analysed during the current study.

## Footnotes

1 The suffix *-scope* comes from the Greek word *skopion*, which means “to observe” or “to look at”, and is used to form words that denote instruments designed for viewing objects (e.g. microscope, telescope).

## Declarations

### Supplementary Information

STL and original design files (AutoDesk 123D Design) for 3D printing the parts of the chlorophyll fluoroscope and of the LED driver housing are provided as supplementary material at….
