# Supplementary figures and images for: The chlorophyll fluoroscope, a device to observe the in vivo emission of chlorophyll fluorescence for teaching and demonstration purposes
Source: Photosynth Res. 2025 Apr 15;163(3):26. doi: 10.1007/s11120-025-01150-9 (PMC12000186; doi:10.1007/s11120-025-01150-9)

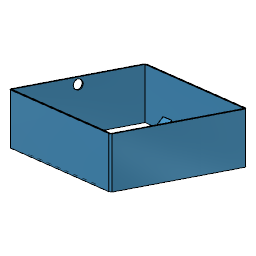

Supplement: Supplementary file 7 — Supplementary Material 7 [file 11120_2025_1150_MOESM7_ESM.123dx › Fusion[Active]/Previews/small.png]

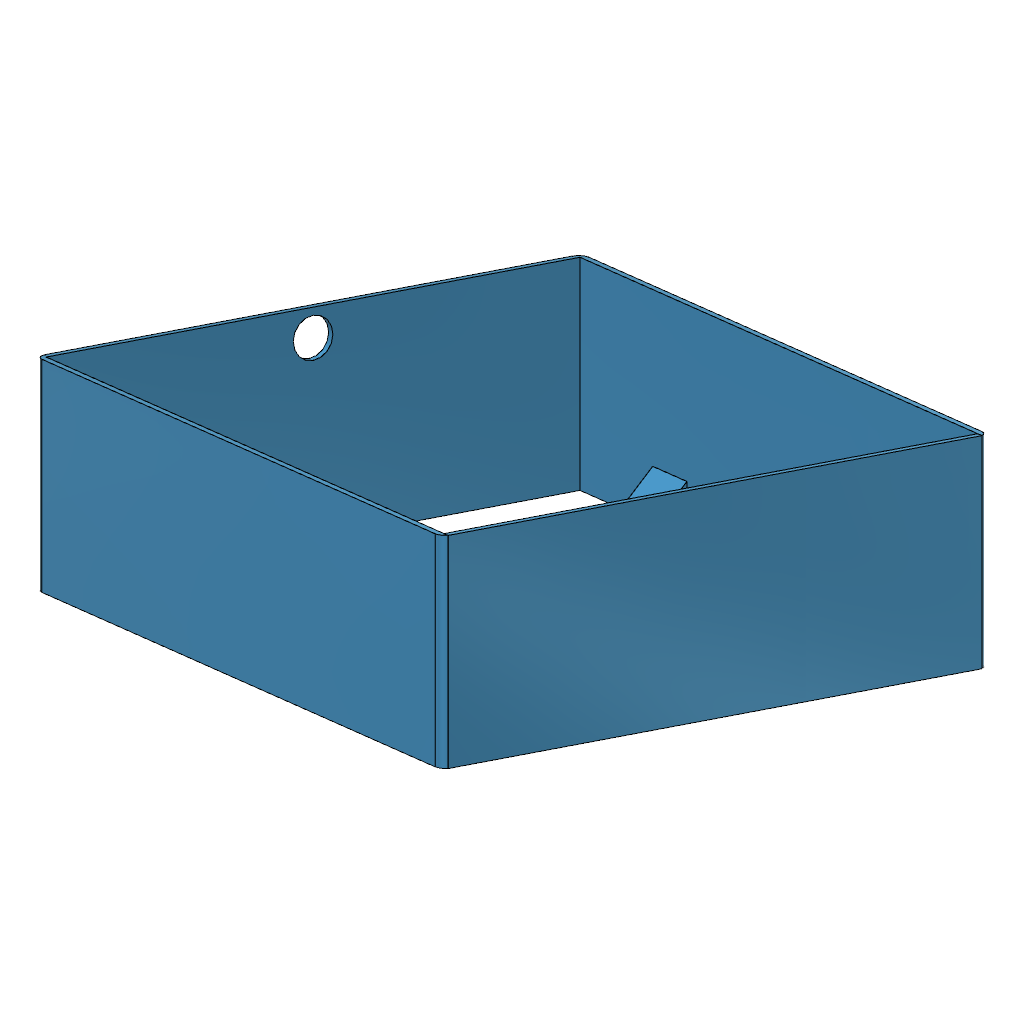

Supplement: Supplementary file 7 — Supplementary Material 7 [file 11120_2025_1150_MOESM7_ESM.123dx › Fusion[Active]/Previews/big.png]

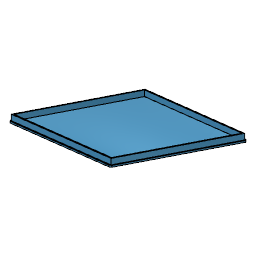

Supplement: Supplementary file 8 — Supplementary Material 8 [file 11120_2025_1150_MOESM8_ESM.123dx › Fusion[Active]/Previews/small.png]

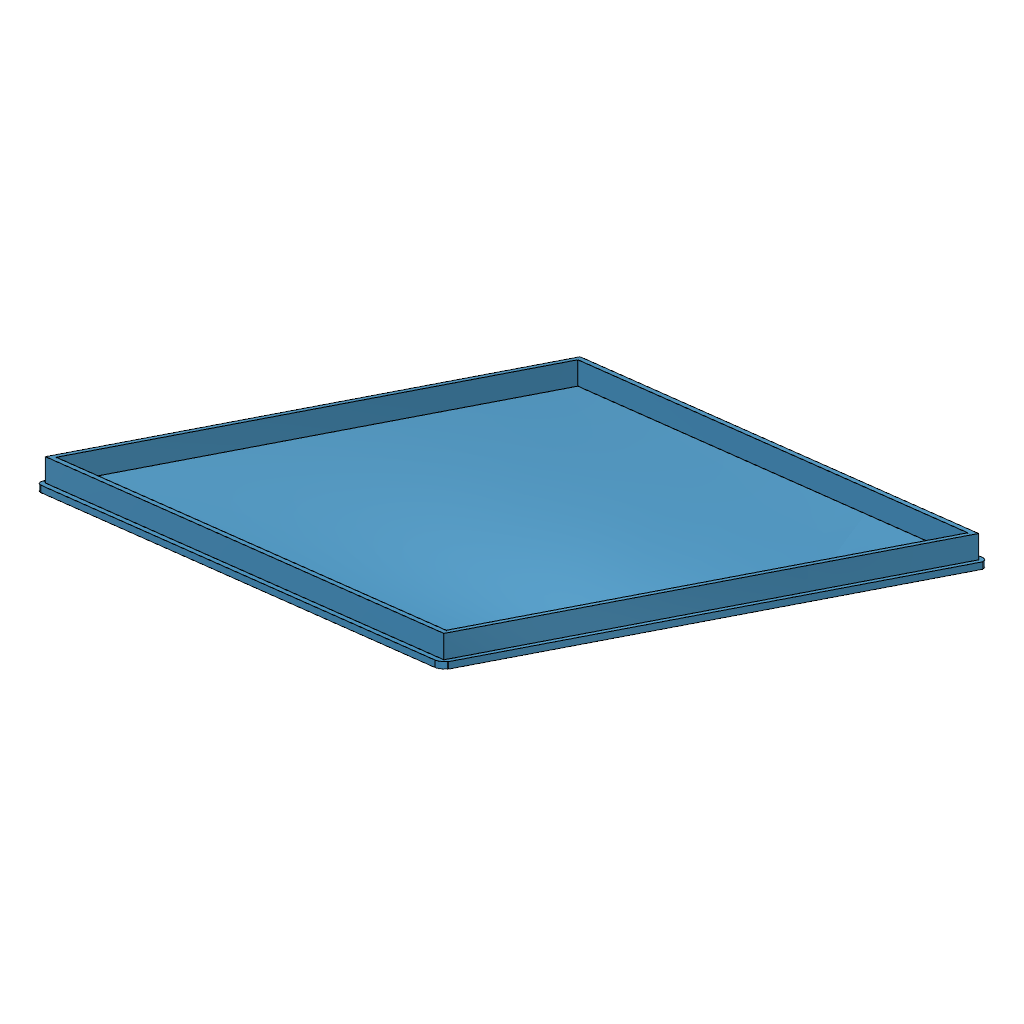

Supplement: Supplementary file 8 — Supplementary Material 8 [file 11120_2025_1150_MOESM8_ESM.123dx › Fusion[Active]/Previews/big.png]

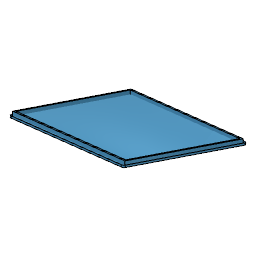

Supplement: Supplementary file 9 — Supplementary Material 9 [file 11120_2025_1150_MOESM9_ESM.123dx › Fusion[Active]/Previews/small.png]

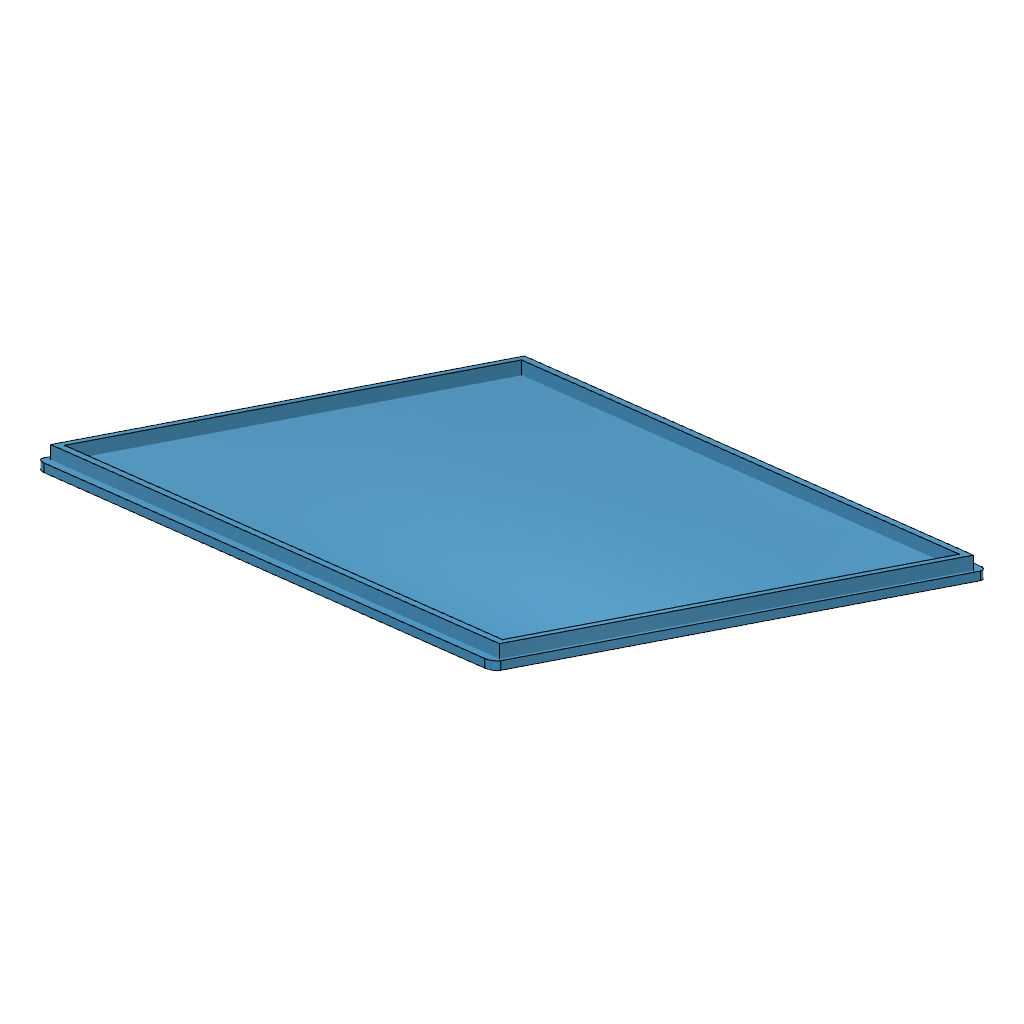

Supplement: Supplementary file 9 — Supplementary Material 9 [file 11120_2025_1150_MOESM9_ESM.123dx › Fusion[Active]/Previews/big.png]

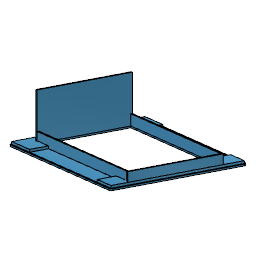

Supplement: Supplementary file 10 — Supplementary Material 10 [file 11120_2025_1150_MOESM10_ESM.123dx › Fusion[Active]/Previews/small.png]

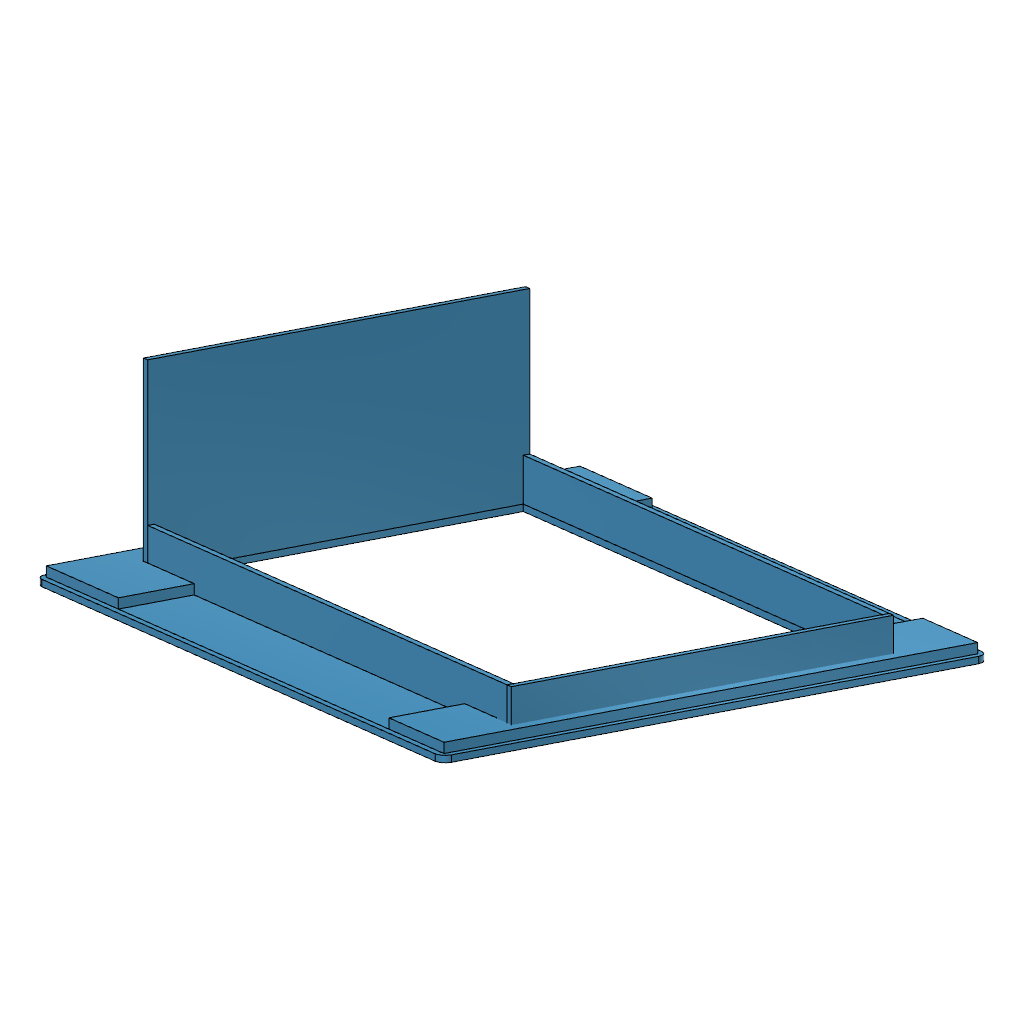

Supplement: Supplementary file 10 — Supplementary Material 10 [file 11120_2025_1150_MOESM10_ESM.123dx › Fusion[Active]/Previews/big.png]

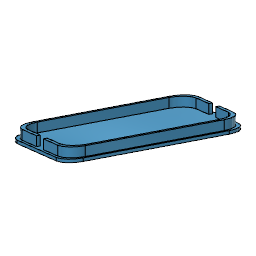

Supplement: Supplementary file 11 — Supplementary Material 11 [file 11120_2025_1150_MOESM11_ESM.123dx › Fusion[Active]/Previews/small.png]

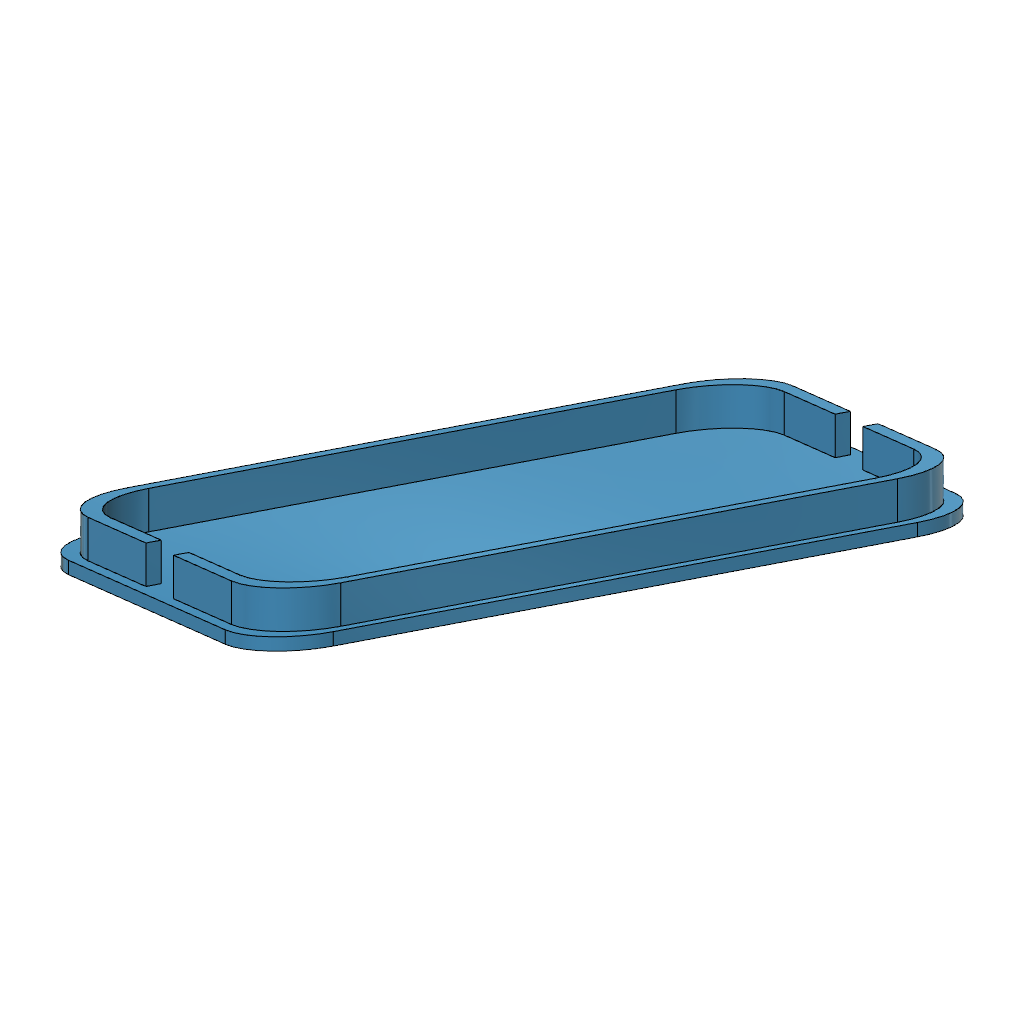

Supplement: Supplementary file 11 — Supplementary Material 11 [file 11120_2025_1150_MOESM11_ESM.123dx › Fusion[Active]/Previews/big.png]

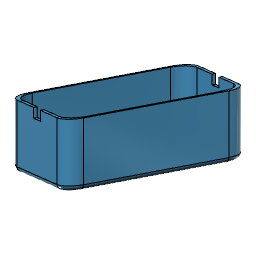

Supplement: Supplementary file 12 — Supplementary Material 12 [file 11120_2025_1150_MOESM12_ESM.123dx › Fusion[Active]/Previews/small.png]

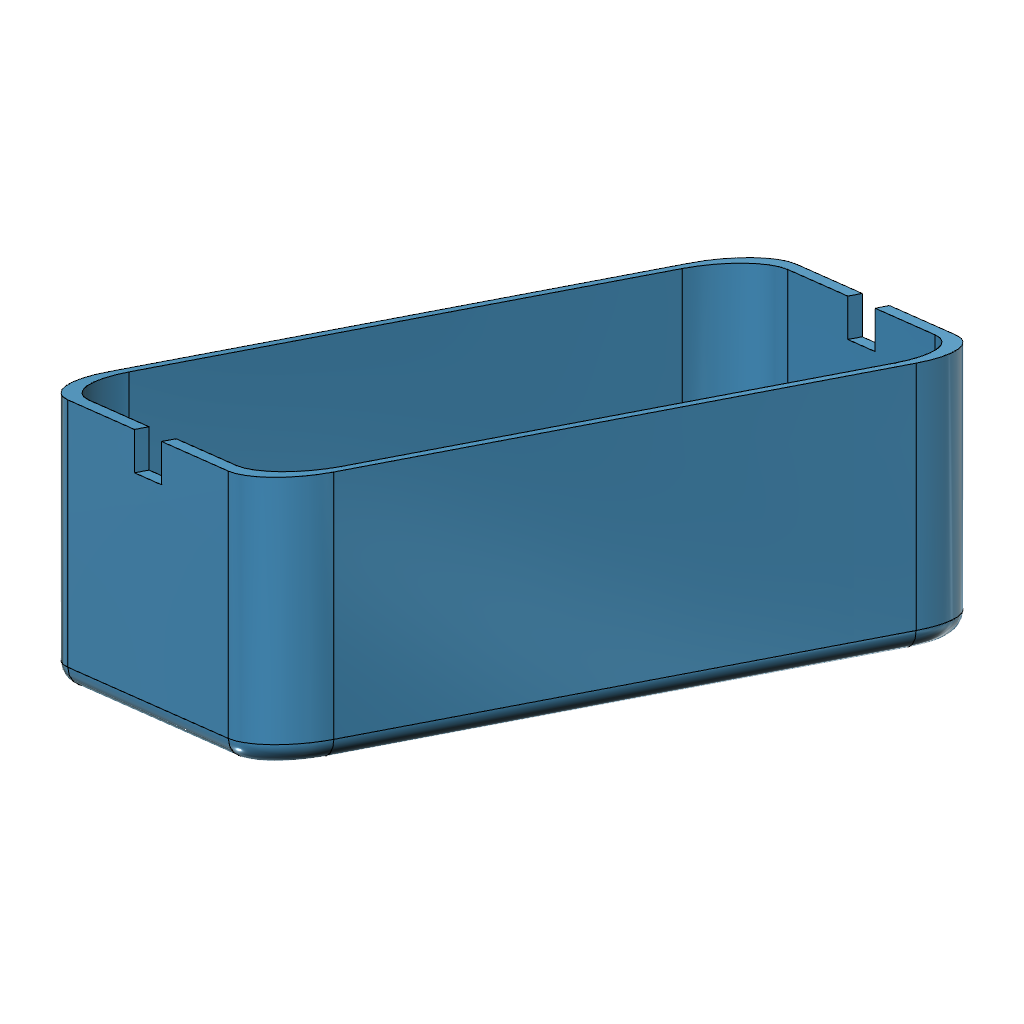

Supplement: Supplementary file 12 — Supplementary Material 12 [file 11120_2025_1150_MOESM12_ESM.123dx › Fusion[Active]/Previews/big.png]
